# Non MRI Guided Accelerated Intermittent Theta Burst Stimulation is Effective in Patients with Treatment Resistant Depression and Suicidality

**DOI:** 10.1192/j.eurpsy.2024.1459

**Published:** 2024-08-27

**Authors:** A. E. Zayed, M. M. Bassiony, U. M. Youssef, G. M. Salah Eldeen, H. M. Elgohary, M. G. Sehlo, O. A. Hefny

**Affiliations:** Psychiatry, Zagazig University, Zagazig, Egypt

## Abstract

**Introduction:**

The U.S. Food & Drug Administration (FDA) has cleared SNT (Stanford Neuromodulation Therapy) for treatment of major depressive disorder (MDD) in adults who have failed to achieve improvement from at least two prior trials of antidepressants. SNT protocol requires both structural and functional connectivity MRIs which is limited by high cost and lack of availability, its use without neuronavigation is still considered an off label use and need more investigation.

**Objectives:**

1-To investigate efficacy of SNT like accelerated off-label protocol without Neuronavigation in treating patients with TRD and suicidality.

2-To investigate durabiliy ( up to one month ) of SNT like accelerated off-label protocol without Neuronavigation in treating patients with TRD and suicidality

**Methods:**

Two cases diagnosed as treatment resistant unipolar depression with suicidal ideations received accelerated intermittent theta burst stimulation (a iTBS); with figure of eight coil administered to the left dorsolateral prefrontal cortex (DL-PFC) determined using Beam method. Stimulation was at 90% MT for 1800 pulses with an intersession interval of fifty minutes. Patients received ten sessions every day for five consecutive days for a total of fifty sessions (90,000 pulses). The following scales were applied at the baseline and at the end of each day of five treatment days:The Montgomery and Asberg Depression Rating Scale (MADRS) The Beck Depression Inventory, Columbia Suicide Severity Rating Scale (C-SSRS) and Young Mania Rating Scale (YMRS).

**Results:**

The two cases at the end of the fifth day were completely improved regarding both suicidal ideations and depression without emerging of hypomania. Follow up was done weekly for one month with durable results.

**Conclusions:**

SNT protocol without neuronavigation needs to be well investigated in suppressing both suicidality and depression in patients with TRD.

**Disclosure of Interest:**

None Declared

